# The Intersectionality of Ageism

**DOI:** 10.1093/geroni/igaa057.3105

**Published:** 2020-12-16

**Authors:** Paul Nash, Tonya Taylor, Becca Levy

## Abstract

Ageism is unlike any other form of prejudice in that, all things being equal, we are all at risk of experiencing it as we age. Whilst this stands true, many communities experience discrimination in other domains such as gender, race, sexuality and disability. As these communities age they are likely to experience cumulative or intersectional disadvantage due to multiple stigmatized group memberships. Where it is understood that there are negative health (cognition, gait speed, longevity, cardiovascular) and psychological (depression, stress, anxiety) outcomes associated with each form of discrimination, the often focused research is less than conclusive regarding the consequences of intersectional discrimination. Using both qualitative and quantitative methodologies from across the US, this symposium explores the impacts of ageism with regard to HIV status, disability status, race/ethnicity and LGBT identification. Exploring the intersections of ageism, each paper will highlight the challenges faced by the respective communities as well as some of the ways to address this using policy, practice and highlighting where additional research would be needed to fully understand the phenomena. Finally the discussant will summarise and collate ideas for a concluding panel discussion.

